# A retrospective audit of young adults who received permanent pacemakers at a teaching hospital in the Western Cape, South Africa

**DOI:** 10.3389/fcvm.2023.1235197

**Published:** 2023-09-29

**Authors:** Elrike Hugo, Anton Doubell, Jan Steyn, Jane Moses

**Affiliations:** Division of Cardiology, Department of Medicine, Faculty of Medicine and Health Sciences, Stellenbosch University, Tygerberg Hospital, Cape Town, South Africa

**Keywords:** pacemaker, atrioventricular block, heart block, mobitz type 1, aetiology, young pacemaker recipients

## Abstract

**Introduction:**

While most pacemaker implantations occur in older individuals, younger patients also receive pacemakers. In these, degenerative conduction system disease is less likely to be the cause of atrioventricular block (AVB), with other diseases being more common. There is, however, a paucity of data on this group as well as on younger pacemaker recipients that have undergone pacemaker implantation for reasons other than AVB. The aim of this study was to perform an audit of young adult permanent pacemaker recipients.

**Method:**

This was a retrospective record review, conducted in the Division of Cardiology at Tygerberg Hospital, Cape Town, South Africa. We included 169 adult patients between the ages of 18 and 60, who received permanent pacemakers between 2010 and 2020. A subgroup analysis of patients 55 years and younger was also performed.

**Results:**

Third degree AVB was the most common indication for pacemaker implantation (*n* = 115; 68%), followed by high degree AVB (*n* = 23; 13.6%) and sick sinus syndrome (SSS; *n* = 14; 8.3%). A specific underlying cause for conduction system abnormalities was found in only 25.4% of patients (*n* = 43), with most of them being 55 years or younger (*n* = 32; 30.8% of patients ≤ 55 years). Specific causes that were identified included prosthetic valve implantation and/or valve repair (*n* = 14; 8.3%), myocardial infarction (*n* = 6; 3.6%), cardiac sarcoidosis (*n* = 5; 3.0%), coronary artery bypass grafting (*n* = 3; 1.8%), cardiomyopathy (*n* = 2; 1.2%), muscular dystrophy (*n* = 2; 1.2%), congenital heart disease (ventricular septal defect; atrioventricular septal defect; Tetralogy of Fallot; bicuspid aortic valve; *n* = 6; 3.6%), acute myocarditis (*n* = 1; 0.6%), atrial myxoma removal (*n* = 1; 0.6%), planned AV node ablation (*n* = 2; 1.2%), and following a previous stab in the chest (*n* = 1; 0.6%).

**Conclusion:**

Given that the mean age of our study population was high, the low number of identified underlying causes in the whole cohort (≤60 years) may reflect some AVB due to age related degeneration of the conductions system in the patients 56 to 60 years age, but also raises the possibility that these patients may be less likely to be extensively investigated for an underlying cause than those ≤55 years, where diseases such as sarcoidosis were more readily confirmed. As access to advanced diagnostic tools improves, the percentage of young pacemaker recipients with an underlying cause identified may increase.

## Introduction

Atrioventricular block (AVB) is the most common indication for permanent pacemaker implantation (1–4). Individuals requiring permanent pacemakers are generally older (3, 5, 6) with a mean age between 64 and 77 (5). This is attributed to the fact that ageing is associated with cardiac conduction system fibrosis (1, 4).

In younger individuals and/or healthy athletes, some degree of heart block may reflect mainly high vagal tone rather than a disease process in the conduction system (7, 8). In other younger patients, a conduction defect may reflect disease processes other than fibrosis, e.g., sarcoidosis (1, 4). There is, however, little data on the progression of disease in this specific patient group, raising the possibility that some younger patients with conduction system disease may not suffer the same risk as older patients with degenerative conduction disease. This may be particularly relevant in young patients with Mobitz 1 AVB, where the current recommendation to consider permanent pacing in individuals as young as 45 years, is largely based on a single study performed in the United Kingdom (9). More data is needed on this subgroup in general, but also specifically for the local population.

The aim of this study was to perform an audit of first time adult permanent pacemaker recipients that were 60 years or younger at first implant with a subgroup analysis of those ≤55 year of age. The objectives were to determine their clinical profile, the indication for permanent pacemaker implantation, the underlying pathology if known, and the pacing need/frequency for those who did not have complete AVB. We set out to better the understanding of which underlying conditions are common in our patient population to inform an appropriate work-up for younger adults presenting with conduction system disease. We also hoped to better understand the disease progression of the young patient with Mobitz 1 AVB.

## Methods

### Study location and population

This was a retrospective record review conducted in the Division of Cardiology at Tygerberg Hospital, Cape Town, South Africa. Ethics approval for the study, which included a waiver of written informed consent, was provided by the Health Research Ethics Committee of the University of Stellenbosch (HREC U21/09/138).

Our study population comprised of permanent pacemaker recipients aged 18 to 60 years, implanted from 2010 to 2020. To be included in the study, patients must have had at least a 6 month follow up and the indication for permanent pacemaker implantation must have been available. Patients that only received a temporary pacemaker and patients with a non-bradycardia indication for device implantation (cardiac resynchronization therapy and implantable cardioverter defibrillators) were excluded from the study. To align with publications utilizing an age cut-off of <55 for young pacemaker recipients we also performed a pre-specified subgroup analysis of this age group.

Data was collected by reviewing the files of adult permanent pacemaker patients, including the Tygerberg Hospital electronic patient records (ECM) and electronic electrocardiogram (ECG) database.

For each patient, their age at first implant, sex, comorbidities, the type of bradyarrhythmia they presented with, the indication for permanent pacemaker implantation, the type of permanent pacemaker implanted, the duration post implant (follow-up period), the pacing need of these patients (expressed as a percentage pacing) at follow-up, the diagnostic imaging modalities utilised and the underlying cause of the conduction system defect, if known, were recorded. The investigations done, type of pacemaker implanted as well as their follow-up were all at the discretion of the attending cardiologist, in accordance with the established guidelines that were available at the time (10–13).

### Statistical analysis

For descriptive purposes frequencies and percentages were reported for categorical variables. Means, standard deviations and medians were reported for continuous measurements. Categorical variables were compared using the Fishers' exact test. Cross tabulation with the Chi-square test was used to test the relationship between age groups and whether a specific underlying cause was identified. Statistical significance was noted if the *p*-value was less than 0.05.

## Results

**Figure 1:**
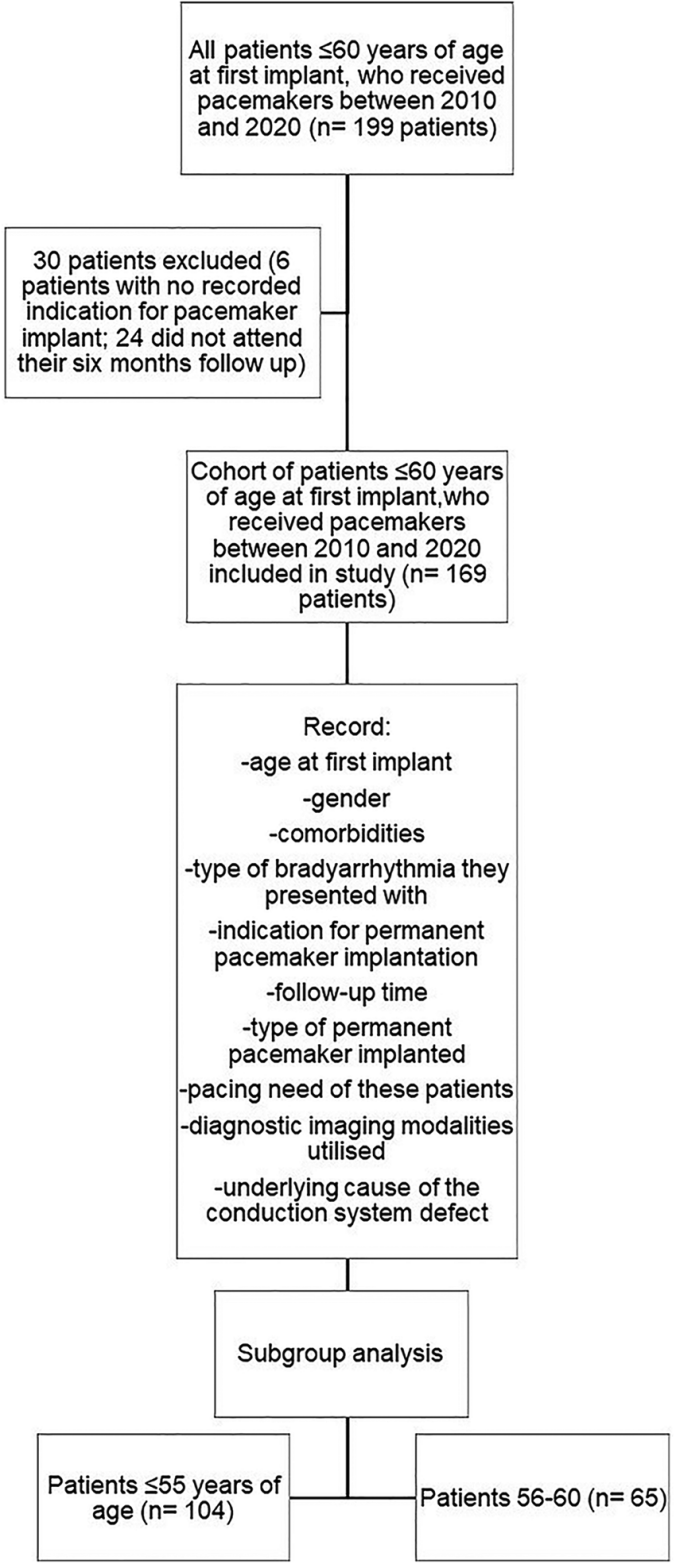
Study layout.

After reviewing the patient information and applying our in- and exclusion criteria, 169 patients were included in the study. Thirty patients were excluded; 6 with no recorded indication for pacemaker implant and 24 that did not attend their six months follow up (Figure 1).

The mean age of our study population was 50.72 (±8.93) years. Fifty three percent of patients (*n* = 90) were female. More than half of our study population (*n* = 104; 61.5%) received their pacemaker at, or before, the age of 55. In the subgroup of 55 years and younger, the mean age was 46.07 (±8.49) years.

Third degree AVB was the most common indication for pacemaker implantation (*n* = 115; 68%), followed by high degree AVB (*n* = 23; 13.6%) and sick sinus syndrome (SSS; *n* = 14; 8.3%). A summary of all the indications for permanent pacemaker implantation can be seen in Table 1.

**Table 1:** Indications for permanent pacemaker implant.

Indication	*n* (%)
Third degree AVB	115 (68%)
High degree AVB (intermittent third degree AVB/2:1 AVB)	23 (13.6%)
SSS	14 (8.3%)
Mobitz type 1	6 (3.6%)
Mobitz type 2	6 (3.6%)
Atrial fibrillation in view of AV nodal ablation	2 (1.2%)
Other^a^	3 (1.8%)
**Total**	**169**

AVB, atrioventricular block; SSS, sick sinus syndrome; AV, atrioventricular; BBB, bundle branch block.

^a^ First degree AVB with alternating left and right BBB (*n* = 1; 0.6%); bifascicular block, second degree AVB (Mobitz type 1) when exercising (*n* = 1; 0.6%), trifascicular block (*n* = 1; 0.6%).

Amongst the patients who presented with third degree AVB, 49 patients (42.6%) had a narrow complex escape, 45 patients (39.1%) had a broad complex escape, 1 patient had a junctional escape with underlying right bundle branch block (RBBB), 2 patients had a junctional escape with underlying bifascicular block and 2 patients had no escape post cardiac surgery, requiring permanent pacing.

Fourteen patients presented with SSS (8.3% of the cohort). Amongst the patients who had SSS, 6 (42.9%) presented with sinus pauses and 3 (21.4%) with tachycardia-bradycardia syndrome. No underlying causes for SSS were identified in this cohort.

Two patients (1.2% of the cohort) presented with atrial fibrillation with a rapid ventricular response and received a pacemaker in view of planned AV nodal ablation.

The subgroup analysis of patients 55 years or younger at the time of implant produced similar results. The most common indication was also third degree AVB (*n* = 72; 69.2% of the subgroup). The other indications for permanent pacemaker implantation in this subgroup included high degree AVB (*n* = 13; 12.5% of the subgroup), second degree AVB Mobitz type 2 (*n* = 2; 1.9% of the subgroup), second degree AVB Mobitz type 1 (*n* = 6; 5.8% of the subgroup), trifascicular block (*n* = 1; 1% of the subgroup) and SSS (*n* = 10; 9.6% of the subgroup).

The majority of patients received a dual chamber pacemaker (*n* = 113; 66.9%) with the most common pacing mode being DDD (*n* = 84; 49.7%).

**Figure 2:**
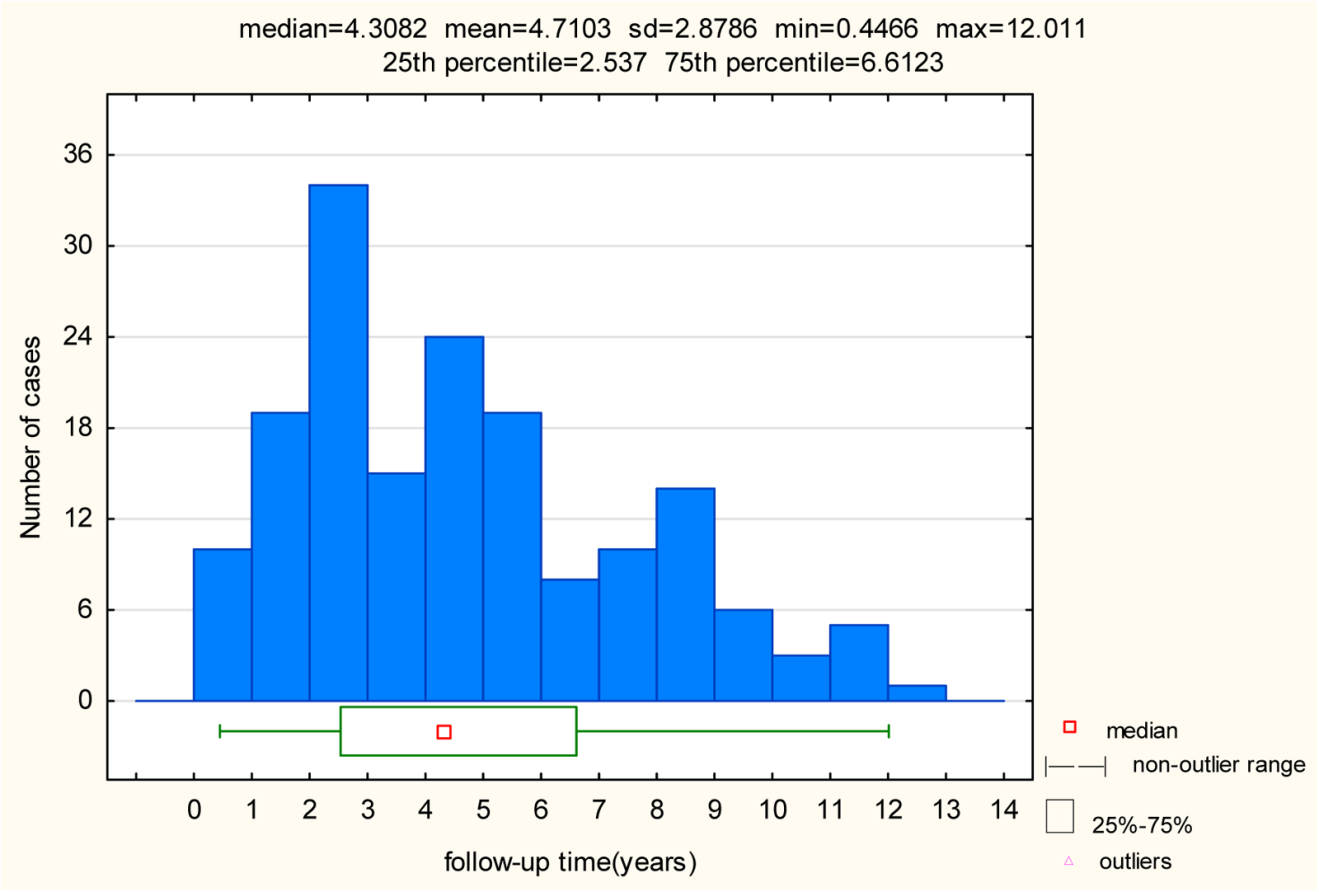
Follow-up time post pacemaker implant.

The mean follow-up time amongst the study population was 4.71 years (±2.88) and the median follow-up time amongst the study population 4.31 years (±2.88). The longest follow-up was 12 years. The overall spread of follow-up times amongst our study population can be seen in Figure 2. Data reported on pacing mode and percentage were taken from the last follow-up visit for each patient.

Overall, 44.4% of patients (*n* = 75) were 100% ventricular paced, the mean ventricular pacing percentage was 75.5% and the median ventricular pacing percentage was 99.0% The mean ventricular pacing percentages were 87.3% for patients with third degree heart block, 64.5% with high degree AVB, 64.7% with second degree AVB Mobitz type 2, 45.4% with second degree AVB Mobitz type 1, and 19.6% with SSS. The patient who had first degree AVB with alternating left and right BBB had a ventricular pacing percentage of 19%, the patient with a bifascicular block and second degree AVB Mobitz type 1 when exercising had a ventricular pacing percentage of 99.9% and the patient with trifascicular block had a ventricular pacing percentage of 98%. The mean atrial pacing percentage for those with SSS was 33.4%.

In the subgroup of patients 55 years and younger, the right ventricular pacing percentages were 84.8% for patients with third degree AVB, 75.2% with high degree AVB, 48.5% with second degree AVB Mobitz type 2, 45.4% with second degree AVB Mobitz type 1 and 14.5% with SSS. In the subgroup of patients between the age 56 and 60, the right ventricular pacing percentages were 91.5% for patients with third degree AVB, 50.5% with high degree AVB, 72.8% with second degree AVB Mobitz type 2, 32.3% with SSS and 52% with atrial fibrillation in view of AV nodal ablation. The patient who had first degree AVB with alternating left and right BBB and the patient with a bifascicular block and second degree AVB Mobitz type 1 when exercising were both older than 55 years of age.

Overall, 23 patients (13.6%) required less than 5% ventricular pacing as indicated by the diagnostic pacemaker parameters at their last follow-up, amongst which 14 patients (8.3%) were 55 years and younger. Amongst the patients 55 years and younger requiring less than 5% right ventricular pacing, 4 patients received their pacemaker for third degree AVB, 1 for high degree AVB, 1 patient for second degree Mobitz type 2, 1 patient for second degree AVB Mobitz type 1 and 7 for SSS.

All of the patients who received their permanent pacemaker for second degree AVB Mobitz type 1 were ≤55 of age at first implant. Half of these patients were male (*n* = 3). The mean right ventricular pacing percentage of 45.4% mentioned above therefore only refers to patients ≤ 55 of age. Only one patient with Mobitz type 1 had a pacing percentage of more than 95% at their last follow-up and only one patient had a right ventricular pacing percentage of less than 5%. All the other patients had some degree of pacing. For 4 out of the 6 patients (66.7% of those with Mobitz type 1), no underlying cause for conduction system abnormalities were identified. Identifiable causes for patients with Mobitz type 1 included valve replacement and/or valve repair (*n* = 1; 16.7%) and Tetralogy of Fallot (*n* = 1; 16.7%).

The majority of patients received a transthoracic echocardiogram (TTE; *n* = 125; 74%) during their hospital stay. Seventy four patients (43.8%) also received a diagnostic chest x-ray, 24 patients (14.2%) a cardiac magnetic resonance (CMR) imaging scan and 7 patients (4.1%) a positron emission tomography (PET) scan during their clinical work-up. A specific cause for the conduction disease was identified in eleven patients in whom CMR imaging was performed (45.8% of patients undergoing CMR imaging) and 4 patients who had a PET scan (57.1% of patients undergoing PET scans).

A specific underlying cause was identified in 25.4% of patients (*n* = 43). This was more likely in the younger subgroup with 32 out of the 43 patients (74.4%) in which an underlying cause was identified, being 55 years and younger and the other 11 (25.6%) being between the age of 56 and 60 (*p* = 0.04).

Specific causes that were identified included prosthetic valve implantation and/or valve repair (*n* = 14; 8.3%), myocardial infarction (*n* = 6; 3.6%), cardiac sarcoidosis (*n* = 5; 3.0%), coronary artery bypass grafting (*n* = 3; 1.8%), cardiomyopathy (*n* = 2; 1.2%), muscular dystrophy (*n* = 2; 1.2%), congenital heart disease (*n* = 6; 3.6%), acute myocarditis (*n* = 1; 0.6%), atrial myxoma removal (*n* = 1; 0.6%), planned AV node ablation (*n* = 2; 1.2%), and following a previous stab in the chest (*n* = 1; 0.6%). Of the 6 patients with congenital heart disease, 3 occurred late post-operative [ventricular septal defect (VSD); atrioventricular septal defect (AVSD); Tetralogy of Fallot (TOF)] and 3 occurred early post-operative (bicuspid aortic valve; AVSD with mitral valve abnormality; TOF). A summary of underlying causes of conduction system defects identified in patients 55 years and younger and those between 56 and 60 can be seen in Table 2.

**Table 2:** Underlying causes of conduction system defects identified in patients ≤ 55 vs. 56–60.

Underlying cause identified	Total cohort (*n* = 169)	Patients ≤ 55 years of age (*n* = 104)	Patients 56–60 years of age (*n* = 65)	*p*-value
Valve replacement and/or repair	14 (8.3%)	12 (11.5%)	2 (3.1%)	0.08
Myocardial infarction	6 (3.6%)	2 (1.9%)	4 (6.2%)	0.21
Sarcoidosis	5 (3.0%)	5 (4.8%)	0 (0%)	0.16
CABG	3 (1.8%)	3 (2.9%)	0 (0%)	0.29
Cardiomyopathy	2 (1.2%)	1 (1.0%)	1 (1.5%)	1.0
Muscular dystrophy	2 (1.2%)	1 (1.0%)	1 (1.5%)	1.0
Congenital heart disease	6 (3.6%)	5 (4.8%)	1 (1.5%)	0.41
Acute myocarditis	1 (0.6%)	1 (1.0%)	0 (0%)	1.0
Atrial myxoma removal	1 (0.6%)	1 (1.0%)	0 (0%)	1.0
Following a previous stab in the chest	1 (0.6%)	1 (1.0%)	0 (0%)	1.0
Planned AV node ablation	2 (1.2%)	0 (0%)	2 (3.1%)	0.15
**Total**	**43**	**32**	**11**	

CABG, coronary artery bypass grafting; AV, atrioventricular.

## Discussion

Globally, pacemaker recipients are generally older, reflecting the fact that ageing is associated with fibrosis of the cardiac conduction system. Assuming similar pathology in younger patients, especially from demographic areas where there is little published data, may result in the search for an underlying cause in young people with conduction disease (e.g., sarcoidosis) being incomplete or inadequate. Underlying causes identified in our study population were not dissimilar from cohorts reported from high income countries. A notable difference is the number of patients receiving pacemakers following valve replacements for rheumatic heart disease.

Some investigations required, such as CMR imaging or PET scans are costly with limited availability in our resource limited environment, and this study emphasizes the value of performing comprehensive investigations for an underlying cause in younger patients requiring pacemaker implantation, particularly in patients ≤ 55 years. The varying pacing requirements in younger individuals with Mobitz 1 AVB in our cohort suggests that in some of these patients, pacemaker implantation may not be required, but this group will require further study to improve the selection of patients with Mobitz 1 AVB that can be managed conservatively.

Given that the mean age of our cohort was 50.72 (±8.93) years, there were some similarities with cohorts not limited by an age cut-off of 60 years. Almost 40% of our cohort was in the 56 to 60 year range, possibly explaining these similarities. In the subgroup of 55 years and younger, the mean age was 46.07 (±8.49). Other studies conducted on young pacemaker recipients who received permanent pacemakers for AVB had a lower mean age, i.e., between 38 and 41 years (1, 2, 4). Women accounted for 54.1% of our study population which is in keeping with a study conducted on cardiac pacing in a referral service in sub-Saharan Africa (14). The relatively high age of our cohort of “young pacemaker recipients” must be taken into account when reflecting on the relatively low number of patients in whom a specific underlying cardiac pathology requiring pacing was identified.. It is likely that a number of patients, even in our age-defined cohort, received pacemakers for AVB due to degenerative changes in the conduction system.

In our study, the majority of patients receiving pacemakers presented with third degree AVB, including those patients ≤ 55 years. This has been shown to be the case for patients across the age spectrum (6, 14–18) and in young cohorts presenting with AVB (1, 4).

The median ventricular pacing percentage of our study population was 99%. The fact that some patients required only 0%–5% ventricular pacing, raises the possibility that some of these younger patients might not have required permanent pacing. Patients with SSS had low ventricular pacing percentages, as expected, since these patients would mainly require atrial pacing. Although the mean right ventricular pacing percentage of those with Mobitz type 1 was not high, the majority of patients required some degree of pacing, with one patient being more than 95% paced. This may support the need to pace younger patients with Mobitz type 1, as found by Shaw et al. (9), but may also reflect pacemaker settings promoting rather than limiting RV pacing, and suggests the need for a further investigation of young patients with Mobitz 1 AVB in order to better the understanding of disease progression.

In our study, a specific cause for the conduction abnormality was found in 25.4% of patients. This was slightly higher in the subgroup ≤ 55 years (*n* = 32; 30.8% of the subgroup). From literature, it is however apparent that this low yield may not be unique to our study population (1, 2, 4). It is important to note that the usual practice in our hospital was to exclude all reversible causes. This includes stopping beta-blocker therapy in patients presenting with heart block and re-evaluating prior to pacemaker implantation.

The most common cause identified in our study was prior cardiac surgery. This was mainly following prosthetic valve implantation and/or valve repair. Other disease states identified were myocardial infarction, cardiac sarcoidosis, muscular dystrophy, congenital heart disease and acute myocarditis. Although the studies conducted by Rudbeck-Resdal et al. (1) and Mkoko et al. (4) included a slightly younger population receiving pacemakers due to AVB, their findings were similar to ours. The most common underlying cause of the AVB in their studies was also following cardiac surgery (1, 4). Similar to our findings, surgical valve replacement was also the main cause of AVB in one of these studies (4). Other underlying causes that have been previously described included congenital heart disease, radiofrequency ablation, cardiomyopathy, muscular dystrophy, ischemic heart disease, sarcoidosis and myocarditis (1). We identified similar causes in our cohort. In our cohort the majority of patients in whom an underlying cause was identified were 55 years and younger. This raises the question whether the patient group between 56 and 60 were not scrutinized sufficiently, or whether these patients had age related degeneration of their conduction system. The most common identified underlying cause in the patients between 56 and 60 years of age was myocardial infarction whereas all the cases caused by sarcoidosis were in the subgroup ≤ 55 years. These findings support the notion that the underlying causes of conduction system defects differ between these age groups of patients. Post-operative heart block following the surgical correction or repair of congenital heart disease has been well described (19–22). In our study population, three patients with congenital heart disease had immediate post-operative heart block which did not revert back to their pre-operative rhythm requiring permanent pacemaker implantation during their time of admission. Three other patients had a delayed presentation of heart block, also requiring permanent pacemaker implantation. Although AVB usually develops at the time or shortly after corrective surgeries, late-onset AVB, as seen in our cohort, also occurs (20, 23).

In our cohort, no patients were recorded to have congenital AVB. This is in contrast to the findings of other studies done on young pacemaker recipients (1, 4). This might be attributed to incomplete record keeping or due to the fact that some patients with congenital heart block might have received permanent pacemakers before the age of 18 and were therefore excluded from our study population.

It is important to note that cardiac imaging modalities such as CMR and PET scans were not readily available at the onset of the study period which may have had an effect on the number of patients in which a specific underlying cause were identified. Cardiac PET scans became available in June 2014 while CMR scans only became available in March 2017. CMR and PET scans are recommended for most patients younger than 60 years of age presenting with AVB to increase the likelihood of identifying the underlying cause (4). Mkoko et al. indicated that CMR imaging has especially been shown to be important diagnostic tool in identifying cardiac sarcoidosis (4). In our study, 45% of those who received CMR imaging and 57% of patients who received a PET scan had an underlying cause identified, suggesting that these are helpful investigations in young patient with conduction disease. As access to these advanced diagnostic tools improves, it will become important to utilize them in the most cost-effective way to improve the diagnostic capabilities in young patients with conduction system disease.

## Limitations

Retrospective studies have inherent limitations. These may include incomplete and/or missing patient data and the inability to collect additional information from patients. An additional limitation was that advanced imaging modalities such as CMR and PET scans were not readily available from the start of the study period, which may have affected the number of patients in which a specific underlying cause was identified.

## Conclusion

In this retrospective cohort, the most common indication for pacemaker implantation was third degree AVB, followed by high degree AVB and SSS. A specific underlying cause was found in 25.4% of patients of which prosthetic valve implantation and/or valve repair was the most common.

Since the mean age of our study populations was high at 50.95 (±8.72) years, we cannot exclude the possibility that some of these patients, especially those between the ages of 55 and 60, might have developed conduction defects as a result of degeneration of their conduction system. The low number of underlying causes identified in patients between the ages of 56 and 60 also raises the possibility that patients in this age bracket are less likely to be extensively investigated for an underlying cause in comparison to those 55 years or younger. As access to advanced diagnostic tools improves, the percentage of young pacemaker recipients with an underlying cause identified may however increase.

Although the mean right ventricular pacing percentage of those with Mobitz type 1 was not high, the majority of patients required some degree of pacing. The reason for this is unclear from this study as it could be due to disease progression or due to pacemaker programming. Further investigation of young patients with Mobitz 1 AVB is needed in order to better the understanding on the predictors of disease progression.

In young patients requiring permanent pacemaker implantation, particularly if they are ≤55 years, we should have a low threshold to recommend augmenting standard echocardiographic imaging with advanced imaging such as CMR/PET scans to identify specific underlying causes. Further prospective studies that include adolescents and young adults are needed to provide more comprehensive and robust data and to improve on our ability to detect underlying causes and risk factors for disease progression in patient with second degree block.

## Data Availability

The raw data supporting the conclusions of this article will be made available by the authors, without undue reservation.
